# Alterations of plant architecture and phase transition by the phytoplasma virulence factor SAP11

**DOI:** 10.1093/jxb/ery318

**Published:** 2018-08-28

**Authors:** Shu Heng Chang, Choon Meng Tan, Chih-Tang Wu, Tzu-Hsiang Lin, Shin-Ying Jiang, Ren-Ci Liu, Ming-Chen Tsai, Li-Wen Su, Jun-Yi Yang

**Affiliations:** 1 Graduate Institute of Biochemistry, National Chung Hsing University, Taichung, Taiwan; 2 PhD Program in Microbial Genomics, National Chung Hsing University and Academia Sinica, Taichung, Taiwan; 3 Bachelor Program of Biotechnology, National Chung Hsing University, Taichung, Taiwan; 4 Graduate Institute of Biotechnology, National Chung Hsing University, Taichung, Taiwan; 5 Advanced Plant Biotechnology Center, National Chung Hsing University, Taichung, Taiwan

**Keywords:** CIN-TCP, effector, miR172/AP2, miR156/SPL, phytoplasma, SAP11, TB/CYC-TCP

## Abstract

As key mediators linking developmental processes with plant immunity, TCP (TEOSINTE-BRANCHED, CYCLOIDEA, PROLIFERATION FACTOR 1 and 2) transcription factors have been increasingly shown to be targets of pathogenic effectors. We report here that TB/CYC (TEOSINTE-BRANCHED/CYCLOIDEA)-TCPs are destabilized by phytoplasma SAP11 effectors, leading to the proliferation of axillary meristems. Although a high degree of sequence diversity was observed among putative SAP11 effectors identified from evolutionarily distinct clusters of phytoplasmas, these effectors acquired fundamental activity in destabilizing TB/CYC-TCPs. In addition, we demonstrate that miR156/SPLs and miR172/AP2 modules, which represent key regulatory hubs involved in plant phase transition, were modulated by Aster Yellows phytoplasma strain Witches’ Broom (AY-WB) protein SAP11. A late-flowering phenotype with significant changes in the expression of flowering-related genes was observed in transgenic Arabidopsis plants expressing SAP11_AYWB_. These morphological and molecular alterations were correlated with the ability of SAP11 effectors to destabilize CIN (CINCINNATA)-TCPs. Although not all putative SAP11 effectors display broad-spectrum activities in modulating morphological and physiological changes in host plants, they serve as core virulence factors responsible for the witches’ broom symptom caused by phytoplasmas.

## Teaser

Phytoplasma SAP11 effectors acquired fundamental activity in destabilizing TB/CYC-TCPs, the key factors controlling axillary meristem development, and serve as core virulence factors responsible for the witches’ broom symptom.

## Introduction

Shoot apical meristems produce different types of organs, such as leaves, branches, and flowers, to initiate distinct developmental phases, for example, the vegetative phase and the reproductive phase (Huijser and Schmid, 2011; Poethig, 2013). Transitions between these developmental phases depend on the physiological state of plants—controlled by various endogenous and environmental cues—which is important for better impact on fitness (Wang and Wang, 2015). However, in plants infected with phytoplasmas, the regulation of developmental processes is disturbed and multiple morphological abnormalities are produced (Lee *et al.*, 2000; Sugio *et al.*, 2011*b*). These phytoplasma-induced alterations in plant architecture, for example, enhanced proliferation of axillary meristems and delayed conversion of the vegetative state to the initiation of flowering, are mainly caused by manipulation of meristem fate (Wei *et al.*, 2013). As has been proposed, the increased generation of vegetative tissues, together with the non-reproductive flower-like structures, over an extended period may be advantageous for the fitness of phytoplasmas and their insect vectors (Sugio *et al.*, 2011*b*; MacLean *et al.*, 2014).

Studies on phytoplasma-secreted virulence factors have identified several effectors that play important roles in modulating the morphological changes of host plants (Sugio *et al.*, 2011*b*). Among them, TENGU, secreted by Onion Yellows phytoplasma strain Mild (OY-M), and SAP11, secreted by Aster Yellows phytoplasma strain Witches’ Broom (AY-WB), have been shown to enhance the proliferation of axillary meristems, which is responsible for the witches’ broom symptom observed in phytoplasma-infected plants (Hoshi *et al.*, 2009; Sugio *et al.*, 2011*a*). Although the molecular mechanisms of TENGU- and SAP11-induced proliferation of axillary meristems remain unknown, small peptides released from the N-terminal of TENGU and its homologs cause morphological changes in host plants (Sugawara *et al.*, 2013). In addition to their effect on the proliferation of axillary meristems, TENGU and SAP11 down-regulate the synthesis of jasmonic acid (JA), resulting in flower sterility and weakening the plant’s defense against insect vectors (Sugio *et al.*, 2011*a*; Minato *et al.*, 2014). Direct plant targets for TENGU have not yet been identified, but SAP11 and its homologs bind to and destabilize class II CIN (CINCINNATA)-TCP (TEOSINTE-BRANCHED, CYCLOIDEA, PROLIFERATION FACTOR 1 and 2) transcription factors, leading to the suppression of JA synthesis (Sugio *et al.*, 2011*a*, 2014).

The TCP gene family encodes plant-specific transcription factors, which serve as evolutionarily conserved mediators of morphological innovations, stress adaptions, and immune responses (Li, 2015; Lopez *et al.*, 2015; Nicolas and Cubas, 2016). TCP transcription factors are categorized into two groups, known as class I and class II, based on the TCP domain (Cubas *et al.*, 1999). Class II TCP transcription factors can be further divided into CIN and TB/CYC (TEOSINTE-BRANCHED/CYCLOIDEA) subgroups. In Arabidopsis, the CIN-TCP subgroup is composed of eight genes (TCP2, TCP3, TCP4, TCP5, TCP10, TCP13, TCP17, and TCP24), which are mainly involved in regulating leaf development, floral organ development, flowering time, and JA-dependent senescence and defense (Palatnik *et al.*, 2003; Efroni *et al.*, 2008; Schommer *et al.*, 2008; Liu *et al.*, 2017; Lucero *et al.*, 2017). In contrast, the Arabidopsis TB/CYC subgroup consists of only three genes (TCP1, TCP12, and TCP18), which have mainly been implicated in controlling axillary meristem development (Aguilar-Martínez *et al.*, 2007; Finlayson, 2007). The crinkled leaves observed in SAP11 transgenic lines are consistent with the findings that members of the CIN-TCP transcription factors are targets of SAP11 and its homologs (Sugio *et al.*, 2014; Tan *et al.*, 2016; Janik *et al.*, 2017). However, it remains unknown whether SAP11 and its homologs can destabilize TB/CYC-TCP transcription factors, leading to the overproduction of axillary meristems.

In addition to changes in plant architecture, phytoplasmas also disturb the developmental phase transitions in plants. For example, bolting and flowering were suppressed in AY-WB phytoplasma-infected Arabidopsis under short-day conditions (MacLean *et al.*, 2011). Moreover, analysis of phytoplasma-responsive microRNAs (miRNAs) revealed the up-regulation of miR156 and down-regulation of miR172 in association with mulberry yellow dwarf disease (Gai *et al.*, 2014). These two miRNAs act as major regulators in determining flowering time by targeting *SQUAMOSA PROMOTER BINDING PROTEIN* (*SBP*)*-LIKE* (*SPL*) genes and *APETALA2* (*AP2*)-type genes (Huijser and Schmid, 2011; Teotia and Tang, 2015). *SPL* genes encode plant-specific transcription factors, which were identified on the basis of their ability to bind a promoter sequence element of the floral meristem identity gene *SQUAMOSA* (Klein *et al.*, 1996). In Arabidopsis, a subset of *SPL* genes is targeted by miR156; these genes can be divided into two classes based on the size of the proteins they encode (Cardon *et al.*, 1999; Guo *et al.*, 2008). Among them, *SPL3*, *SPL4*, and *SPL5* encode small proteins that serve as accelerators of phase transition and promote flowering through the direct activation of *LEAFY* (*LFY*), *FRUITFULL* (*FUL*), and *APETALA1* (*AP1*) (Yamaguchi *et al.*, 2009). Conversely, the expression of miR156 declines gradually in an age-dependent manner and leads to the reproductive phase transition (Wang *et al.*, 2009; Wu *et al.*, 2009). Thus, the miR156/SPLs regulatory module serves as a key hub in determining the timing of phase transition and flowering (Huijser and Schmid, 2011; Wang and Wang, 2015). Similar to the miR156/SPLs module, the miR172/AP2 module plays a role in regulating phase transition and flowering (Aukerman and Sakai, 2003). However, downstream of miR156, miR172 displays an antagonistic expression pattern to miR156 and targets *AP2*-type genes, which act as floral repressors (Teotia and Tang, 2015; Wu *et al.*, 2009). Although it remains unknown how the developmental phase transitions in plants are altered by phytoplasmas, a significant decrease in miR172 was observed in *SAP11* transgenic plants (Lu *et al.*, 2014).

Further analysis of SAP11 effectors is necessary to provide new insights into the molecular mechanisms underlying the pathogenesis of phytoplasmas. In this study, SAP11 and its putative homologs identified from AY-WB phytoplasma, *Candidatus* Phytoplasma mali (CaPM), Peanut witches’ broom (PnWB) phytoplasma, and OY-M phytoplasma, named SAP11_AYWB_, SAP11_CaPM_, SAP11_PnWB_, and SAP11_OYM_, respectively, were further characterized. Specifically, we assessed their functions in the alteration of plant architecture and phase transition.

## Materials and methods

### Plant materials and growth conditions

*Arabidopsis thaliana* ecotype Col-0 grown at 21 °C was used to generate transgenic lines and obtain protoplasts. *Nicotiana benthamiana* grown at 26 °C was used for transient expression assays. Plants were grown in semi-controlled walk-in chambers under a 16 h light/8 h dark photoperiod to measure flowering time and count branching numbers, as previously described (Aguilar-Martínez *et al.*, 2007; Liu *et al.*, 2017).

### Generation of transgenic Arabidopsis plants

To enhance bacterial protein expression *in planta*, synthetic DNA with codon-optimized sequences encoding SAP11 effectors without signal peptides were amplified by PCR using AccuPrime pfx DNA polymerase (Invitrogen) and subcloned into the binary vector pBA002 (Kost *et al.*, 1998) for expression under the control of a cauliflower mosaic virus (CaMV) 35S promoter. The freeze-thaw method was performed to introduce plasmid DNA into *Agrobacterium tumefaciens* strain ABI. *Agrobacterium*-mediated transformation of Arabidopsis using the floral dip method was performed to generate transgenic plants (Zhang *et al.*, 2006). Transgenic Arabidopsis expressing SAP11_AYWB_, SAP11_CaPM_, SAP11_PnWB_, and SAP11_OYM_ were screened on 0.5× Murashige and Skoog medium containing Basta (25 μg ml^−1^) and carbenicillin (100 μg ml^−1^). Homozygous transgenic lines examined by western blotting using specific antibodies were utilized for further studies.

### Polyclonal antibody production and western blotting

Codon-optimized DNA fragments encoding SAP11 effectors without the signal peptide were subcloned into the SUMO-pET vector and introduced into *Escherichia coli* BL21 (DE3). N-terminal His-SUMO-tagged SAP11 proteins were expressed and purified by Ni^2+^-NTA resin (Qiagen) according to the manufacturer’s instructions. Then, the proteins were cleaved with ubiquitin-like-specific protease 1 to remove the His-SUMO tag. Recombinant SAP11 effectors obtained using a Sephacryl S-200 HR gel filtration column (GE Healthcare) were prepared for polyclonal antibody production in rabbits. For western blotting, SAP11_AYWB_ was detected using anti-SAP11_AYWB_ serum at 1:10000 dilution, SAP11_CaPM_ was detected using anti-SAP11_CaPM_ serum at 1:2500 dilution, and SAP11_PnWB_ and SAP11_OYM_ were detected using anti-SAP11_PnWB_ serum at 1:10000 dilution. Amersham ECL reagents were used. Chemiluminescence signals were captured with an ImageQuant LAS 4000 mini imager (GE Healthcare).

### Co-expression assays

Arabidopsis *TCP* genes and *Oryza sativa TB1* were amplified from cDNA libraries synthesized with SuperScript III First-Strand Synthesis SuperMix (Invitrogen) according to the manufacturer’s instructions. DNA fragments subcloned into the binary vector pBA-N-SFP (Su *et al.*, 2013) for expression under the control of a CaMV 35S promoter were transformed into *A. tumefaciens* strain ABI. SAP11 effectors and N-terminal FLAG-tagged TCP transcription factors (SFP-TCPs) were co-expressed in *N. benthamiana* leaves by agroinfiltration (Leuzinger *et al.*, 2013) using a mixture of *A. tumefaciens* carrying the desired constructs. After 2 days, leaves were collected and ground into powder after freezing with liquid nitrogen. Then, total cell extracts were prepared by directly adding 0.2 ml 2.5× SDS sample buffer (5 mM EDTA, 5% SDS, 0.3 M Tris–HCl, pH 6.8, 20% glycerol, 1% β-mercaptoethanol, and bromophenyl blue) to 0.1 g sample powder. The extracts were heated in a boiling water bath for 5 min and then centrifuged at 13000 *g* for 10 min. After centrifugation, the supernatant was obtained and proteins were separated by SDS-PAGE. Specific polyclonal antibodies to SAP11 effectors and monoclonal anti-FLAG™ tag antibody were used to monitor protein amounts. All experiments were repeated at least five times using biologically distinct samples. Each sample was prepared from two infiltrated leaves (the third and fourth leaves, counting from the top of 4- to 5-week-old plants).

### Subcellular localization assays

Codon-optimized DNA fragments encoding SAP11 effectors without a signal peptide were subcloned into the pWEN25 vector (Kost *et al.*, 1998) under the control of a CaMV 35S promoter for transient expression in Arabidopsis mesophyll protoplasts. Protoplasts were isolated from 4- to 5-week-old Arabidopsis leaves digested with Cellulase R10 and Macerozyme R10 (Yakult Pharmaceutical Ind. Co.) as previously described (Yoo *et al.*, 2007). Briefly, after digestion, protoplasts were resuspended in W5 solution [154 mM NaCl, 125 mM CaCl_2_, 5 mM KCl, and 2 mM 2-(*N*-morpholino)ethanesulfonic acid, pH 5.7] and transfected with 10 μg of plasmid DNA using the polyethylene glycol (PEG)–calcium method. After 12 h of incubation, fluorescence signals for N-terminal yellow fluorescent protein (YFP)-tagged SAP11 effectors were observed using a confocal laser scanning microscope, and images were collected with the Olympus Fluoview FV1000 system.

For transient expression in *N. benthamiana* leaves, DNA fragments encoding YFP-tagged SAP11 effectors without signal peptides were subcloned into the binary vector pBA002 and then transformed into *A. tumefaciens* strain ABI for agroinfiltration (Leuzinger *et al.*, 2013). The yellow fluorescence signals were monitored after 36 h post-inoculation using a confocal laser scanning microscope, and images were collected with the Olympus Fluoview FV1000 system. All experiments were repeated at least three times using biologically distinct samples.

### Real-time quantitative reverse transcription PCR

TRIzol^TM^ (Invitrogen)-extracted total RNA from 14-day-old Arabidopsis plants was reverse transcribed using SuperScript III First-Strand Synthesis SuperMix (Invitrogen) according to the manufacturer’s instructions. Briefly, each reverse transcription reaction was performed with 1 μg of total RNA at 50 °C using a mixture of random hexamers and oligo(dT)_20_. PCRs consisting of three technical replicates were performed on a CFX96^TM^ Real-time System (Bio-Rad) using a KAPA SYBR Fast qPCR Kit (Kapa Biosystems) under the following conditions: 95 °C for 1 min followed by 40 cycles of 95 °C for 15 s, 58 °C for 15 s, and 72 °C for 30 s. The reference gene *Actin2* was used to normalize the expression levels of selected genes. All experiments were repeated at least three times using biologically distinct samples. Each sample was prepared from 10 Arabidopsis transgenic plants (the entire plant with roots).

### TaqMan miRNA assay

TRIzol^TM^ (Invitrogen)-extracted total RNA from 14-day-old Arabidopsis was reverse transcribed using a TaqMan MicroRNA Reverse Transcription Kit (Applied Biosystems) according to the manufacturer’s instructions. Briefly, each reverse transcription was performed with 10 ng of total RNA and miRNA-specific stem-loop primer (Applied Biosystems) on a thermocycler under the following conditions: 16 °C for 30 min, 42 °C for 30 min, and 85 °C for 5 min. The individual cDNA was further used for real-time quantitative reverse transcription PCR (qRT–PCR) to quantify the expression of targeted miRNA. For qRT–PCR, TaqMan Universal Master Mix II (Applied Biosystems) and specific TaqMan probes (miR156 ID000333, SnoR85 ID001711) were used to measure the expression level of miRNAs. All measurements were performed in three replicates under the following conditions: 95 °C for 10 min followed by 40 cycles of 95 °C for 15 s and 60 °C for 1 min. The expression level of the small non-coding RNA SnoR85 was used as an internal control for normalizing the data.

### Iodine staining

Starch in rosette leaves was examined by iodine staining as previously described (Bahaji *et al.*, 2011) with the following modifications. Whole plants (14 days old) were harvested and immediately fixed with 3.7% formaldehyde in phosphate buffer for 10 min. Leaf pigments were removed by the addition of 95% ethanol. After rehydration, samples were stained with 80% Lugol solution (Sigma) for 30 min and then rinsed in deionized water until the blue precipitate of starch was distinguishable from the yellowish background.

### Primers

The primer sequences for plasmid constructions and qRT–PCR analyses are listed in Supplementary Table S1 at JXB online.

## Results

### SAP11 effectors are present in a range of phylogenetically distant phytoplasmas

**Fig. 1.**
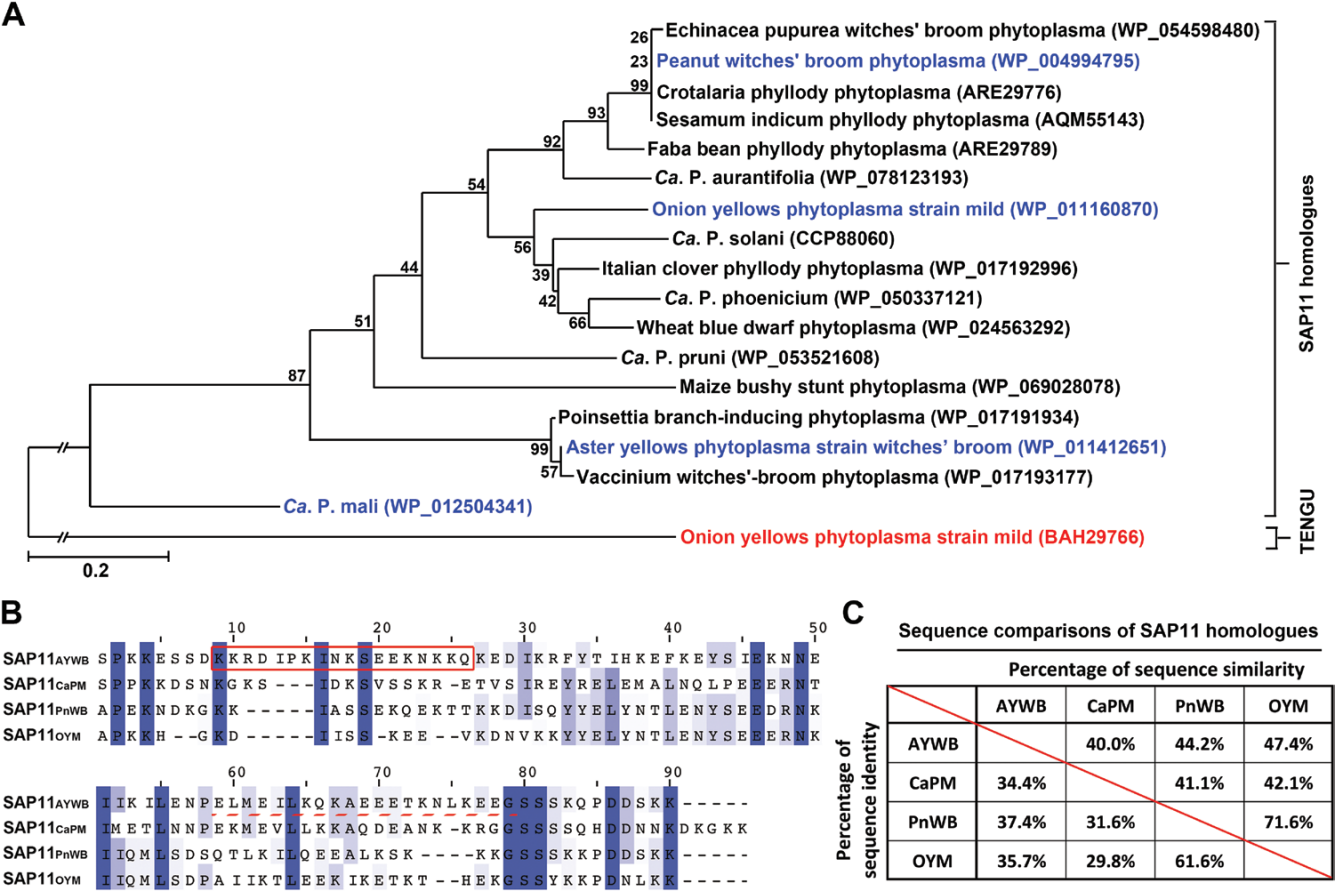
Phylogenetic comparison and sequence alignment of phytoplasma SAP11 effectors. (A) Phylogenetic tree constructed based on the comparison of protein sequences of SAP11 effectors. The neighbor-joining method followed by bootstrap analysis was used with MEGA 7.0 software. TENGU served as an outgroup. The numbers at the branch points are bootstrap values that represent the percentages of replicate trees based on 1000 repeats. (B) Sequence alignment of SAP11 effectors without a signal peptide constructed by MEGA 7.0 using ClustalW and edited by using Jalview software. Identical residues are shaded in blue, and the color gradient indicates the level of sequence conservation at each position. The nuclear localization signal is indicated with a red rectangle, and the coiled-coil domain with a dashed red line. (C) Sequence comparison of SAP11 effectors without a signal peptide. Sequence identity and sequence similarity are presented on the lower left and upper right, respectively.

A BLAST search against the NCBI protein sequence database was performed using SAP11_AYWB_ and SAP11_CaPM_ as queries. Putative homologs of SAP11 were identified in members of the genus *Phytoplasma*, including different 16S rRNA groups (Fig. 1A; Supplementary Fig. S1A). Although these putative homologous proteins displayed diverse protein sequence similarities with one another, several amino acid positions showed a high level of residue conservation (Fig. 1B; Supplementary Fig. S1B). To investigate whether these putative homologous proteins are functional orthologs, SAP11_AYWB_, SAP11_CaPM_, SAP11_PnWB_, and SAP11_OYM_ with protein sequence similarities ranging from 40% to 72% were selected for further characterization (Fig. 1C).

### Phenotypic analyses of transgenic Arabidopsis expressing phytoplasma SAP11 effectors

Arabidopsis T1 transgenic plants expressing SAP11_AYWB_, SAP11_CaPM_, SAP11_PnWB_, or SAP11_OYM_ without a signal peptide were generated under the control of a CaMV 35S promoter. Compared with vector-only transgenic plants, independent *35S::SAP11* transgenic lines exhibited alterations in plant architecture with various degrees of severity (Supplementary Fig. S2; Supplementary Table S2). The homozygous lines expressing individual transgenes were selected and examined by polyclonal antibodies generated against SAP11_AYWB_, SAP11_CaPM_, and SAP11_PnWB_ (Supplementary Fig. S3). Owing to the high sequence similarity between SAP11_OYM_ and SAP11_PnWB_, the expression of SAP11_OYM_ could be readily recognized by the antibody to SAP11_PnWB_.

**Fig. 2.**
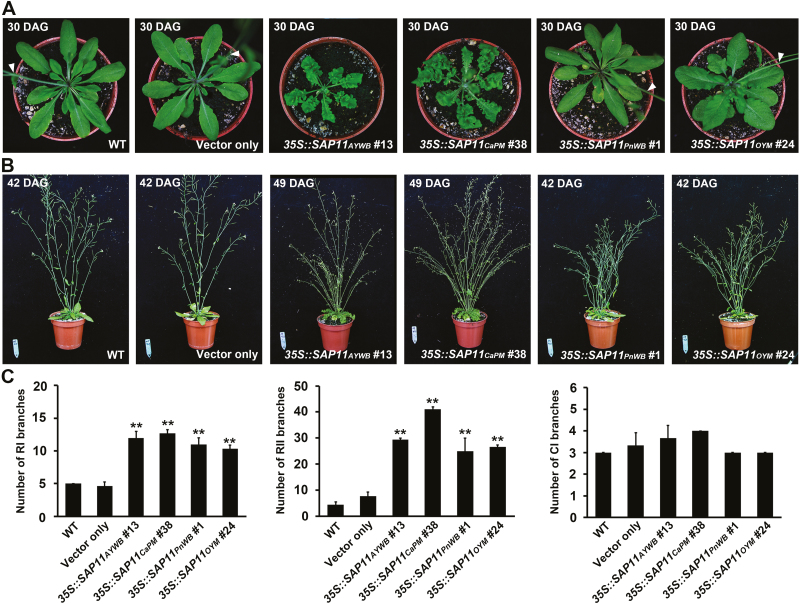
Phenotypic comparison of rosette leaves and branching shoots of Arabidopsis homozygous transgenic lines. (A) Morphological changes in rosette leaves of *35S::SAP11* transgenic plants compared with wild-type (WT) and vector-only transgenic plants. Images were obtained at 30 days after germination (DAG). Arrowheads indicate the inflorescence. (B) Morphological changes in branching shoots of *35S::SAP11* transgenic plants compared with WT and vector-only transgenic plants. Images were obtained at 20 days after flowering. (C) Numbers of primary RI, RII, and CI branches of *35S::SAP11* transgenic plants, WT plants, and vector-only transgenic plants. Statistically significant differences were determined using Student’s *t*-test (**P<0.01 for *35S::SAP11* transgenic plants versus vector-only controls).

The alterations caused by SAP11 effectors were observed in leaf architecture, shoot architecture, and flowering time. Regarding leaf morphology, severe serration and crinkled phenotypes were typically observed in *35S::SAP11*_*AYWB*_ transgenic lines, whereas mild serration and crinkled phenotypes were typically observed in *35S::SAP11*_*CaPM*_ transgenic lines (Fig. 2A; Supplementary Fig. S4A). However, only wild-type (WT) or very weakly altered leaf phenotypes were noted in *35S::SAP11*_*PnWB*_ and *35S::SAP11*_*OYM*_ transgenic lines (Fig 2A; Supplementary Fig. S4A). In addition, an obviously late-flowering phenotype was associated with the *35S::SAP11* transgenic lines that exhibited serrated and crinkled leaves (Fig. 2A; Supplementary Fig. S4A). Notably, regarding shoot morphology, transgenic lines expressing SAP11_AYWB_, SAP11_CaPM_, SAP11_PnWB_, or SAP11_OYM_ usually displayed a bushy phenotype similar to the witches’ broom symptom caused by phytoplasmas (Fig. 2B; Supplementary Fig. S4B). The number of primary rosette-leaf (RI) and secondary rosette-leaf (RII) branches, but not primary cauline-leaf (CI) branches, increased dramatically in all *35S::SAP11* transgenic lines compared with those of WT and vector-only transgenic plants (Fig. 2C; Supplementary Fig. S4C). Taken together, these findings show that the SAP11 effectors tested exhibited a strong ability to enhance the proliferation of axillary meristems. However, leaf morphology and flowering time were altered to varying degrees among the transgenic lines.

### Phytoplasma SAP11 effectors destabilize class II TB/CYC-TCP transcription factors

**Fig. 3.**
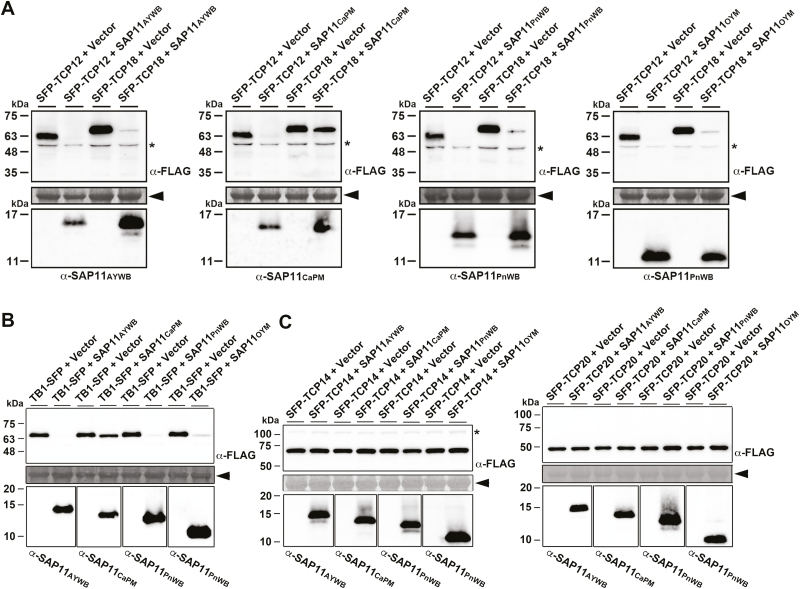
Co-expression assays of SAP11 effector-mediated destabilization of class II TB/CYC-TCP transcription factors. The relative abundance of Arabidopsis and rice class II TB/CYC-TCPs (TCP12, TCP18, and TB1) (A, B) and Arabidopsis class I PCF/CYC-TCPs (TCP14 and TCP20) (C) were examined in the presence of SAP11 effectors through transient co-expression assays in *N. benthamiana*. Western blotting was conducted to examine the expression levels of FLAG-tagged TCPs (upper panel) and SAP11 effectors (lower panel) using a monoclonal antibody to the FLAG tag and polyclonal antibodies to SAP11 effectors. SAP11_OYM_ could be recognized by α-SAP11_PnWB_. As a loading control, the large subunit of Rubisco visualized with Coomassie Brilliant Blue staining is indicated by the arrowhead (middle panel). Non-specific bandings recognized by antibodies are indicated by asterisks.

It was previously demonstrated that SAP11_AYWB_ binds to and destabilizes class II CIN-TCPs, but not class I PCF-TCPs (Sugio *et al.*, 2011*a*). However, previous studies have not characterized whether SAP11 effectors destabilize class II TB/CYC-TCPs, the key factors controlling axillary meristem development. To understand whether the bushy phenotype caused by SAP11 effectors is correlated with their ability to destabilize class II TB/CYC-TCPs, we conducted co-expression assays in *N. benthamiana*. Using agroinfiltration, *35S* promoter-driven cDNAs encoding SAP11 effectors and FLAG-tagged *A. thaliana* or *O. sativa* TB/CYC-TCPs (SFP-TCP12, SFP-TCP18, and TB1-SFP) were co-expressed *in planta*. As a result, most class II TB/CYC-TCPs were absent or greatly decreased in abundance in the presence of SAP11_AYWB_, SAP11_PnWB_, and SAP11_OYM_ compared with the presence of vector alone (Fig. 3A, B). By contrast, SAP11_CaPM_ exhibited a strong ability to destabilize TCP12, but not TCP18 or TB1 (Fig. 3A, B). As a control, the *A. thaliana* class I PCF-TCPs SFP-TCP14 and SFP-TCP20 were not decreased in abundance in the presence of any of the SAP11 effectors compared with the presence of the vector alone (Fig. 3C). These findings are consistent with the highly branching phenotype observed in *35S::SAP11* transgenic lines. Hence, the SAP11-mediated destabilization of TB/CYC-TCPs suggests that SAP11 effectors play a significant role in witches’ broom diseases associated with phytoplasmas.

To provide more evidence, the putative homologs of SAP11—SAP11_WBDP_, SAP11_ICPP_, SAP11_CaPS_, SAP11_VWBP_, and SAP11_CaPPr_, identified from Wheat blue dwarf phytoplasma (WBDP), Italian clover phyllody phytoplasma (ICPP), *Candidatus* Phytoplasma solani (CaPS), Vaccinium witches’ broom phytoplasma (VWBP), and *Candidatus* Phytoplasma pruni (CaPPr), respectively—were examined for their ability to destabilize FLAG-tagged *A. thaliana* TCP12 and TCP18. TCP12 and TCP18 were greatly decreased in abundance in the presence of SAP11_WBDP_, SAP11_ICPP_, SAP11_CaPS_, SAP11_VWBP_, and SAP11_CaPPr_ compared with the presence of the vector alone in co-expression assays (Supplementary Fig. S5).

### Phytoplasma SAP11 effectors exhibit different abilities in destabilizing class II CIN-TCP transcription factors

**Fig. 4.**
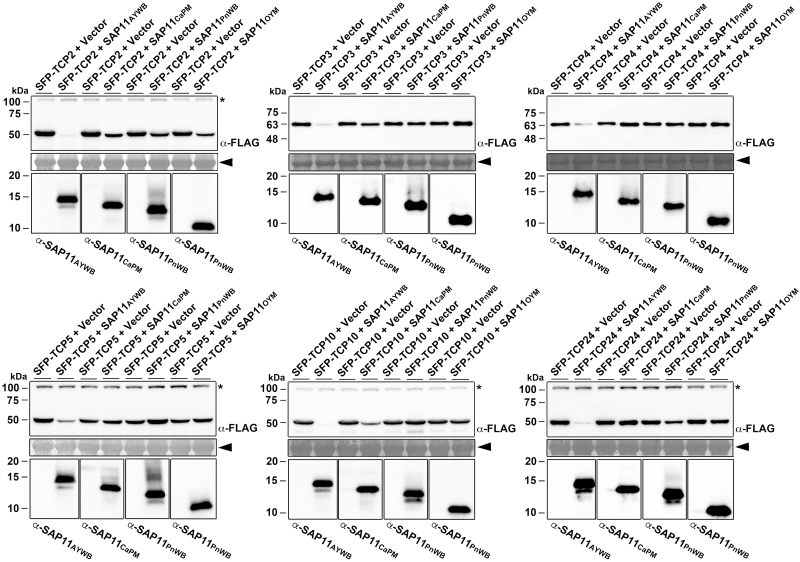
Co-expression assays of SAP11 effector-mediated destabilization of class II CIN-TCP transcription factors. The relative abundance levels of Arabidopsis class II CIN-TCPs (TCP2, TCP3, TCP4, TCP5, TCP10, and TCP24) were examined in the presence of SAP11 effectors through transient co-expression assays in *N. benthamiana*. Western blotting was conducted to examine the expression levels of FLAG-tagged TCPs (upper panel) and SAP11 effectors (lower panel) using a monoclonal antibody to the FLAG tag and polyclonal antibodies to SAP11 effectors. SAP11_OYM_ could be recognized by α-SAP11_PnWB_. As a loading control, the large subunit of Rubisco visualized with Coomassie Brilliant Blue staining is indicated by the arrowhead (middle panel). Non-specific bandings recognized by antibodies are indicated by asterisks.

To understand whether the varying degrees of alteration in leaf shape caused by SAP11 effectors were dependent on their ability to destabilize class II CIN-TCPs, key factors controlling leaf development, 35S promoter-driven cDNAs encoding SAP11 effectors and FLAG-tagged *A. thaliana* CIN-TCPs (SFP-TCP2, SFP-TCP3, SFP-TCP4, SFP-TCP5, SFP-TCP10, and SFP-TCP24) were co-expressed in *N. benthamiana* by agroinfiltration. SAP11_AYWB_ exhibited a strong ability to destabilize most class II CIN-TCPs; SAP11_CaPM_ exhibited a weak ability to destabilize TCP2 and TCP10 but was not able to destabilize TCP3, TCP4, TCP5, and TCP24; and SAP11_PnWB_ and SAP11_OYM_ exhibited only a weak ability to destabilize TCP2 (Fig. 4). These findings are consistent with the results that severe serration and crinkled leaves were typically observed in *35S::SAP11*_*AYWB*_ transgenic lines (Supplementary Fig. S2; Supplementary Table S2).

### Distinct subcellular localization patterns of phytoplasma SAP11 effectors in plants

**Fig. 5.**
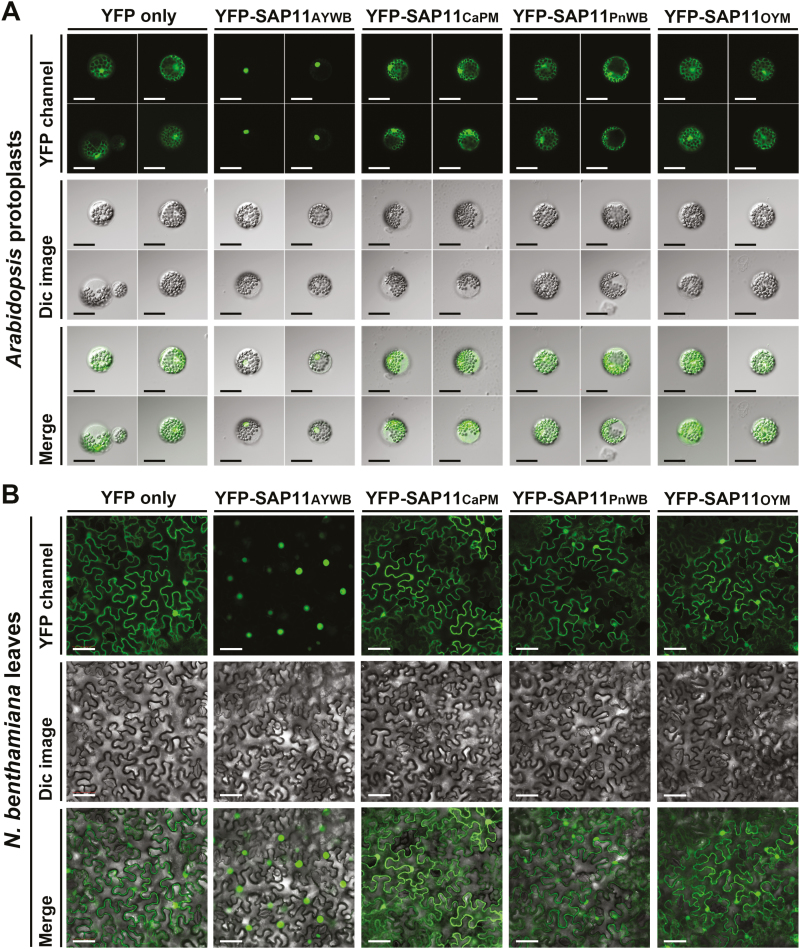
Subcellular localization of SAP11 effectors. N-terminal YFP-fused SAP11 effectors were transiently expressed in Arabidopsis mesophyll protoplasts (A) and *N. benthamiana* leaves (B) under the control of the 35S promoter. Fluorescence signals of YFP-fused SAP11 effectors and YFP only were imaged using a confocal laser scanning microscope and the Olympus Fluoview FV1000 system. Scale bars=40 μm (A), 50 μm (B). Dic, Differential interference contrast.

Bioinformatics analysis using the PSORT program (http://psort.hgc.jp) indicated that only SAP11_AYWB_, and not SAP11_CaPM_, SAP11_PnWB_, or SAP11_OYM_, contains a bipartite nuclear localization signal (NLS) (Fig. 1B). To examine the subcellular localization patterns of SAP11 effectors, N-terminal YFP fusion constructs were generated under the control of the CaMV 35S promoter. YFP and YFP-fused SAP11 effectors were transiently expressed in Arabidopsis mesophyll protoplasts and *N. benthamiana* leaves by PEG transfection and agroinfiltration, respectively. Confocal laser scanning microscopic analysis showed that YFP-SAP11_CaPM_, YFP-SAP11_PnWB_, and YFP-SAP11_OYM_, as well as a YFP-only control, were distributed evenly throughout the cell, whereas YFP-SAP11_AYWB_ displayed a nuclear localization pattern in Arabidopsis mesophyll protoplasts and *N. benthamiana* leaves (Fig. 5A, B; Supplementary Table S3). Examination of protein expression levels by western blotting further demonstrated that the fluorescence images of YFP-fused SAP11 effectors did not result from the cleavage of YFP (Supplementary Fig. S6). As a result, the protein sizes of SAP11 effectors are small enough to diffuse through the nuclear pore to target nuclear-localized TCP transcription factors.

### Phytoplasma SAP11_AYWB_ effector delays flowering time by altering the expression levels of flowering-related genes in Arabidopsis

**Fig. 6.**
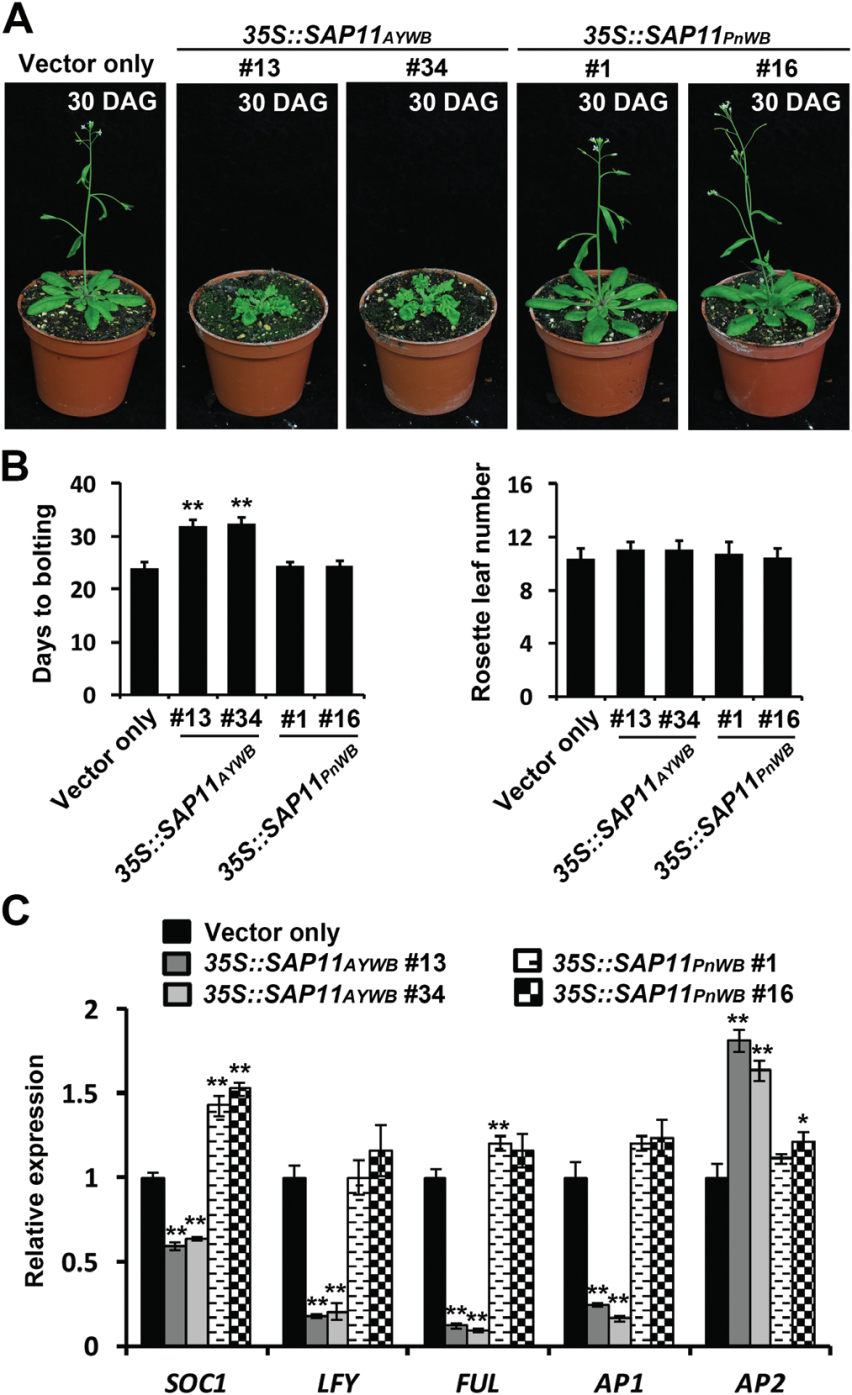
Examination of flowering-related genes and the flowering time of Arabidopsis homozygous transgenic plants. (A) Morphological changes in the flowering of *35S::SAP11* transgenic plants compared with those in vector-only transgenic plants. Images were obtained at 30 days after germination (DAG). (B) Days to bolting and number of rosette leaves at bolting of *35S::SAP11* transgenic plants and vector-only transgenic plants. (C) Transcript levels of flowering-related genes in *35S::SAP11* transgenic plants, examined by qRT–PCR and normalized to *Actin2*. The relative expression level of each gene in vector-only transgenic plants was set to 1. Statistically significant differences were determined using Student’s *t*-test (*P<0.05, **P<0.01 for *35S::SAP11* transgenic plants versus vector-only controls).

To investigate the molecular mechanism of the late-flowering phenotype observed in *35S::SAP11* transgenic lines with serrated and crinkled leaves, *35S::SAP11*_*AYWB*_ transgenic lines were selected for further characterization and compared with *35S::SAP11*_*PnWB*_ transgenic lines, which exhibit no change in leaf phenotype (Fig. 6A). Under the long-day condition used in this study, *35S::SAP11*_*AYWB*_ transgenic lines, but not *35S::SAP11*_*PnWB*_ transgenic lines, displayed an obvious late-flowering phenotype compared with the vector-only transgenic plant (Fig. 6A, B). However, the rosette leaf number at flowering time was not obviously different between the *35S::SAP11* transgenic lines and the vector-only control (Fig. 6B).

We also examined the expression levels of several flowering-related genes, including *SUPPRESSOR OF OVEREXPRESSION OF CO 1* (*SOC1*), *LFY*, *FUL*, *AP1*, and *AP2*, which play important roles in the regulation of flowering time. Using qRT–PCR, we found that SAP11_AYWB_, but not SAP11_PnWB_, significantly repressed the expression of the flowering pathway integrator *SOC1* and the floral meristem-identity genes *LFY*, *FUL*, and *AP1* (Fig. 6C). In addition, SAP11_AYWB_ enhanced the expression of the flowering repressor gene *AP2* (Fig. 6C). These significant changes in the expression levels of flowering-related genes are consistent with the alteration of flowering time in *35S::SAP11*_*AYWB*_ transgenic lines.

### The phytoplasma SAP11_AYWB_ effector modulates phase transition by regulating the miR156/SPLs module and starch metabolism in Arabidopsis

**Fig. 7.**
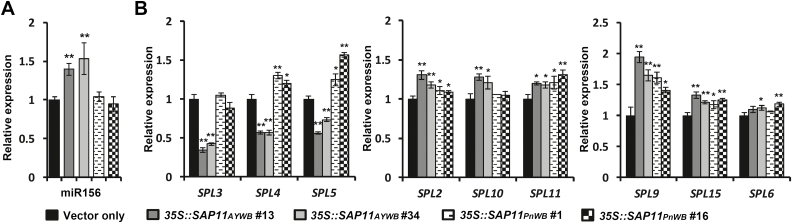
Regulation of the miR156/SPLs module by phytoplasma SAP11_AYWB_. (A) miR156 expression levels in *35S::SAP11* transgenic Arabidopsis plants, examined by qRT–PCR using a TaqMan miRNA assay. The relative expression levels of miR156 in vector-only transgenic plants were set to 1 after normalization to the small non-coding RNA SnoR85. (B) Transcript levels of miR156-targeted *SPL* genes in *35S::SAP11* transgenic plants, examined by qRT–PCR and normalized to *Actin2*. The relative expression level of each gene in vector-only transgenic plants was set to 1. Statistically significant differences were determined using Student’s *t*-test (*P<0.05, **P<0.01 for *35S::SAP11* transgenic plants versus vector-only controls).

AY-WB phytoplasma delays the conversion of the vegetative state to the initiation of flowering (MacLean *et al.*, 2011). To understand whether the late-flowering phenotype observed in *35S::SAP11*_*AYWB*_ transgenic lines was correlated with a delayed phase transition, the miR156/SPLs module, a key regulatory hub in determining phase changes, was investigated. Using TaqMan miRNA assays, we found that the level of miR156 increased significantly in Arabidopsis with the expression of SAP11_AYWB_ but not SAP11_PnWB_ (Fig. 7A). In Arabidopsis, miR156-targeted *SPL* genes can be grouped into four clades: *SPL3*/*SPL4*/*SPL5*, *SPL2*/*SPL10*/*SPL11*, *SPL9*/*SPL15*, and *SPL6*/*SPL13*. Only the gene transcripts of the *SPL3*/*SPL4*/*SPL5* clade decreased consistently and significantly in *35S::SAP11*_*AYWB*_ transgenic lines (Fig. 7B). These results are consistent with the functions of the *SPL3, SPL4*, and *SPL5* genes, which appear to play roles in controlling flowering time and phase change.

**Fig. 8.**
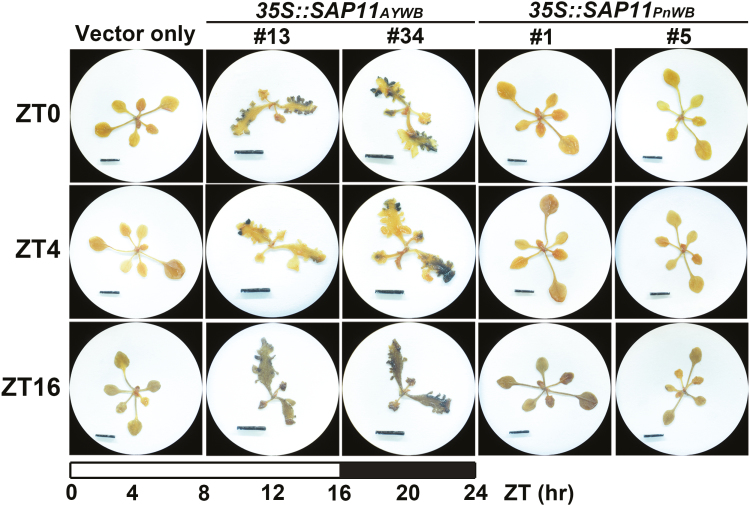
Alteration of starch accumulation by phytoplasma SAP11_AYWB_. The accumulation of starch in *35S::SAP11* transgenic Arabidopsis plants was examined at different times of the day [zeitgeber time (ZT) 0, ZT4, and ZT16] by iodine staining. White and black bars at the bottom of the diagram indicate subjective day and night, respectively.

As an important storage carbohydrate that provides energy for plant growth and plays a role in phase transition (Matsoukas *et al.*, 2013), starch mainly accumulates in leaves during the day and then degrades the following night. We examined the accumulation of starch in Arabidopsis transgenic lines by iodine staining at different times of day. No starch accumulation was observed in *35S::SAP11*_*PnWB*_ or vector-only transgenic lines at the beginning [zeitgeber time (ZT) 0] and at 4 h (ZT4) of the day period (Fig. 8). However, high levels of starch accumulation in the leaf margins of *35S::SAP11*_*AYWB*_ transgenic lines were observed at ZT0 and ZT4 (Fig. 8). These results provide evidence supporting the finding that *35S::SAP11*_*AYWB*_ transgenic lines display a late-flowering phenotype.

## Discussion

In this study, we report for the first time that class II TB/CYC-TCP transcription factors are destabilized by phytoplasma SAP11 effectors. TB/CYC-TCPs that act as negative regulators to control axillary meristem development have been identified in different plant species (Nicolas and Cubas, 2016). Among these TB/CYC-TCPs, TB1/BRC1 is considered a central integrator of multiple endogenous and environmental pathways that regulate axillary bud outgrowth (Rameau *et al.*, 2015; Teichmann and Muhr, 2015). To date, only two phytoplasma-secreted virulence factors, SAP11 and TENGU, have been identified to enhance the proliferation of axillary meristems. However, unlike TENGU effectors, which were cloned only in the 16SrI group (Sugawara *et al.*, 2013; Wang *et al.*, 2014), SAP11 effectors are putatively present in a range of phylogenetically distant phytoplasmas, including the 16SrI, 16SrII, 16SrIII, 16SrIX, 16SrX, and 16SrXII groups (Fig. 1A; Supplementary Fig. S1A). Although a high degree of sequence diversity was identified among SAP11 effectors (Fig. 1B; Supplementary Fig. S1B), these effectors exhibited great ability to destabilize class II TB/CYC-TCPs (Fig. 3A, B; Supplementary Fig. S5). Moreover, transgenic plants expressing SAP11 effectors displayed strong activity in enhancing the proliferation of axillary meristems (Fig. 2C; Supplementary Fig. S4C; Supplementary Table S2) similar to the axillary meristem proliferation in plants infected with AY-WB, CaPM, PnWB, or OY-M phytoplasmas. Thus, our results imply that SAP11 effectors function as a potential core virulence factor for the witches’ broom symptom induced by phytoplasmas.

SAP11_AYWB_ exhibited stronger activity in destabilizing class II CIN-TCPs than SAP11_CaPM_, SAP11_PnWB_, and SAP11_OYM_ (Fig. 4). As a result, severe serration and crinkled leaves were mainly noted in *35S::SAP11*_*AYWB*_ transgenic lines (Supplementary Fig. S2; Supplementary Table S2), and the phenotype is strongly related to CIN-TCP levels in a dose-dependent manner (Nicolas and Cubas, 2016). In Arabidopsis, the class II CIN-TCP subgroup comprises eight genes. Five of these genes, TCP2, TCP3, TCP4, TCP10, and TCP24, are targeted by miR319 (Palatnik *et al.*, 2003). Previous studies have demonstrated that the Arabidopsis jaw-D mutant, which overexpresses miR319, and the tcp4 mutant displayed late-flowering phenotypes (Palatnik *et al.*, 2003; Schommer *et al.*, 2008), indicating the putative role of the miR319/CIN-TCPs module in controlling flowering. The enhanced activity of SAP11_AYWB_ in destabilizing CIN-TCPs is therefore consistent with the finding that *35S::SAP11*_*AYWB*_ transgenic lines displayed an obvious late-flowering phenotype in addition to the severe serration and crinkled leaves (Fig. 6A, B). This alteration may be beneficial for the colonization of phytoplasmas by prolonging the plant’s lifespan.

In addition to the miR319/CIN-TCPs module, we further found that SAP11_AYWB_ delayed flowering by modulating the miR156/SPLs module, a regulatory hub in phase transitions. Remarkably, although almost all miR156-targeted *SPL* genes play a role in accelerating phase transition (Preston and Hileman, 2013), only gene transcripts of the *SPL3*/*SPL4*/*SPL5* clade decreased significantly, which was paralleled by the accumulation of miR156 in *35S::SAP11*_*AYWB*_ transgenic lines (Fig. 7A, B). SPL3/SPL4/SPL5 are relatively small proteins composed of only an SBP DNA-binding domain (Klein *et al.*, 1996). As upstream activators of floral meristem identity genes, SPL3/SPL4/SPL5 bind directly to the promoters of the *LFY*, *FUL*, and *AP1* genes (Yamaguchi *et al.*, 2009). This result is consistent with our findings that the expression levels of *LFY*, *FUL*, and *AP1* were significantly decreased in *35S::SAP11*_*AYWB*_ transgenic lines (Fig. 6C). We previously demonstrated that, unlike the accumulation of miR156, miR172 expression was significantly suppressed in *35S::SAP11*_*AYWB*_ transgenic lines (Lu *et al.*, 2014). We found that the expression of *AP2*, an miR172-targeted gene, was up-regulated in *35S::SAP11*_*AYWB*_ transgenic lines (Fig. 6C). Interestingly, *AP2*, a flowering repressor gene, can further positively regulate miR156 and negatively regulate miR172 in a feedback loop to fine-tune the transition to flowering (Yant *et al.*, 2010). Therefore, the regulation of miR156/SPLs and miR172/AP2 modules by SAP11_AYWB_ provides molecular evidence of phase transition, supporting the findings that bolting and flowering are suppressed in AY-WB phytoplasma-infected Arabidopsis (MacLean *et al.*, 2011).

Although the late-flowering phenotype observed in *35S::SAP11*_*AYWB*_ transgenic lines is potentially mediated through the destabilization of miR319-regulated CIN-TCPs, the molecular mechanism of their actions in regulating flowering time remain largely unknown. In addition, the association with miR156/SPLs and miR172/AP2 modules is unknown. Based on data showing that TCP4 binds in vitro to the conserved TCP binding site located in the upstream region of the SPL3 promoter, it has been suggested that TCP4 regulates flowering via direct activation of SPL3 (Sarvepalli and Nath, 2011). However, recently, TCP4 was found to directly activate the expression of CONSTANS (CO) through binding to its promoter (Kubota *et al.*, 2017; Liu *et al.*, 2017). As a central component in promoting photoperiodic flowering, CO directly activates the transcription of its downstream targeting gene, *FT*, which in turn positively regulates SOC1 (Samach *et al.*, 2000; Suárez-López *et al.*, 2001; Yoo *et al.*, 2005). SOC1 is a flowering pathway integrator that functions as a direct transcriptional activator of *SPL3*/*SPL4*/*SPL5* genes (Jung *et al.*, 2012). Thus, the down-regulation of *SPL3*/*SPL4*/*SPL5* genes observed in *35S::SAP11*_*AYWB*_ transgenic lines might be regulated indirectly by the SAP11_AYWB_-mediated destabilization of TCP4 through SOC1.

In plants, starch is an important storage carbohydrate that provides energy when plants are unable to conduct photosynthesis. Starch is mainly accumulated in leaves during the day and then degraded the following night. In Arabidopsis, mutants defective in starch metabolism display a late-flowering phenotype and a prolonged juvenile phase. For instance, the *starch-excess1* mutant, which is defective in starch degradation, accumulated high amounts of starch and exhibited a delayed flowering time (Matsoukas *et al.*, 2013). Similarly, the *ADP-Glc pyrophosphorylase 1* mutant, which is unable to produce starch, exhibited a late-flowering phenotype (Matsoukas *et al.*, 2013). In the present study, a high starch accumulation phenotype was observed in *35S::SAP11*_*AYWB*_ transgenic lines, even at the end of the dark period. In contrast, no starch accumulation was observed in *35S::SAP11*_*PnWB*_ transgenic lines or the vector-only control (Fig. 8). These data are consistent with the late-flowering phenotype displayed by *35S::SAP11*_*AYWB*_ transgenic lines.

In contrast to AY-WB phytoplasma, which readily infects the model plant Arabidopsis, CaPM phytoplasma causes a severe disease (apple proliferation) in apple trees. The typical symptoms of apple proliferation disease include witches’ broom, chlorotic leaves with enlarged stipules, and late blooming (Rid *et al.*, 2016). In addition, the leaves of infected trees are irregularly serrated and are smaller than normal (Rid *et al.*, 2016). Although it is still unclear whether Arabidopsis can be infected by CaPM phytoplasma, Arabidopsis transgenic plants expressing SAP11_CaPM_ display crinkled leaves (Fig. 2A) and a late-flowering phenotype (data not shown), which are similar to the symptoms that appear in apple proliferation disease. These phenomena are possibly related to the partial activity of SAP11_CaPM_ in destabilizing CIN-TCPs (Fig. 4). Although SAP11 effectors exhibited different activities in destabilizing CIN-TCPs, they acquired fundamental activity in destabilizing TB/CYC-TCPs. These results provide evidence to explain the typical witches’ broom symptom that was apparent in PnWB-infected peanut plants, in which no irregularly serrated leaves were observed (Liu *et al.*, 2015).

Although SAP11 effectors exhibit the ability to enhance the proliferation of axillary meristems, they alter leaf morphology and flowering time to varying degrees (Supplementary Fig. S2; Supplementary Table S2). SAP11 effectors are relatively small proteins, and the NLS motif and TCP-binding coiled-coil domain are the least conserved domains among SAP11 effectors (Sugio *et al.*, 2014). In this study, only SAP11_AYWB_ contained an NLS motif in the N-terminal region (Fig. 1B). Although the NLS motif may increase the likelihood of SAP11_AYWB_ targeting nuclear-localized CIN-TCPs, the SAP11 effectors are likely small enough to diffuse through the nuclear pore (Fig. 5A, B). Thus, the apparent discrepancy in alterations in plant architecture might be caused by the wide diversity of sequences in coiled-coil domains of SAP11 effectors (Fig. 1B). These differences may be responsible for different binding affinities with various CIN-TCPs, leading to the destabilization of CIN-TCPs to varying degrees (Sugio *et al.*, 2014). However, further analysis is required to elucidate the molecular mechanism underlying the regulatory specificity of the SAP11-mediated destabilization of CIN-TCPs.

In addition to the morphological changes of leaf crinkling and shoot branching, SAP11 effectors appear to weaken plant defense responses, induce phosphate starvation responses, modulate the synthesis of volatile organic compounds, and delay flowering time (Sugio *et al.*, 2011*a*; Lu *et al.*, 2014; Tan *et al.*, 2016). Although we did not observe the broad-spectrum activities in modulating plant morphological and physiological changes in all SAP11 effectors, our findings provide evidence to successfully dissect the multiple functions of SAP11 effectors. We have shown that SAP11 effectors exhibit fundamental activity to destabilize class II TB/CYC-TCPs, providing new insights into the witches’ broom symptom caused by phytoplasmas.

## Supplementary data

Supplementary data are available at *JXB* online.

Fig. S1. Phylogenetic comparison and protein sequence alignment of phytoplasma 16Sr RNAs and SAP11 effectors.

Fig. S2. Morphological comparison of independent Arabidopsis T1 transgenic plants.

Fig. S3. Western blotting analysis of Arabidopsis homozygous transgenic lines expressing SAP11 effectors.

Fig. S4. Phenotypic comparison of rosette leaves and branching shoots of Arabidopsis homozygous transgenic lines.

Fig. S5. Co-expression assays of SAP11 effector-mediated destabilization of class II TB/CYC-TCP transcription factors.

Fig. S6. Western blotting analysis of YFP and YFP-fused SAP11 effectors expressed in Arabidopsis mesophyll protoplasts and *N. benthamiana* leaves.

Table S1. Primer sequences for plasmid constructions and qRT–PCR.

Table S2. Summary of the morphological comparison of independent Arabidopsis T1 transgenic plants.

Table S3. Summary of the subcellular localization comparison of SAP11 effectors.

Supplementary Figures

Supplementary Tables
