# Supplementary material for: Alterations of plant architecture and phase transition by the phytoplasma virulence factor SAP11
Source: J Exp Bot. 2018 Aug 28;69(22):5389–401. doi: 10.1093/jxb/ery318 (PMC6255702; doi:10.1093/jxb/ery318)
Supplement: Supplementary Figures [file ery318_suppl_supplementary_figures.pdf]

Supplementary Fig. S1

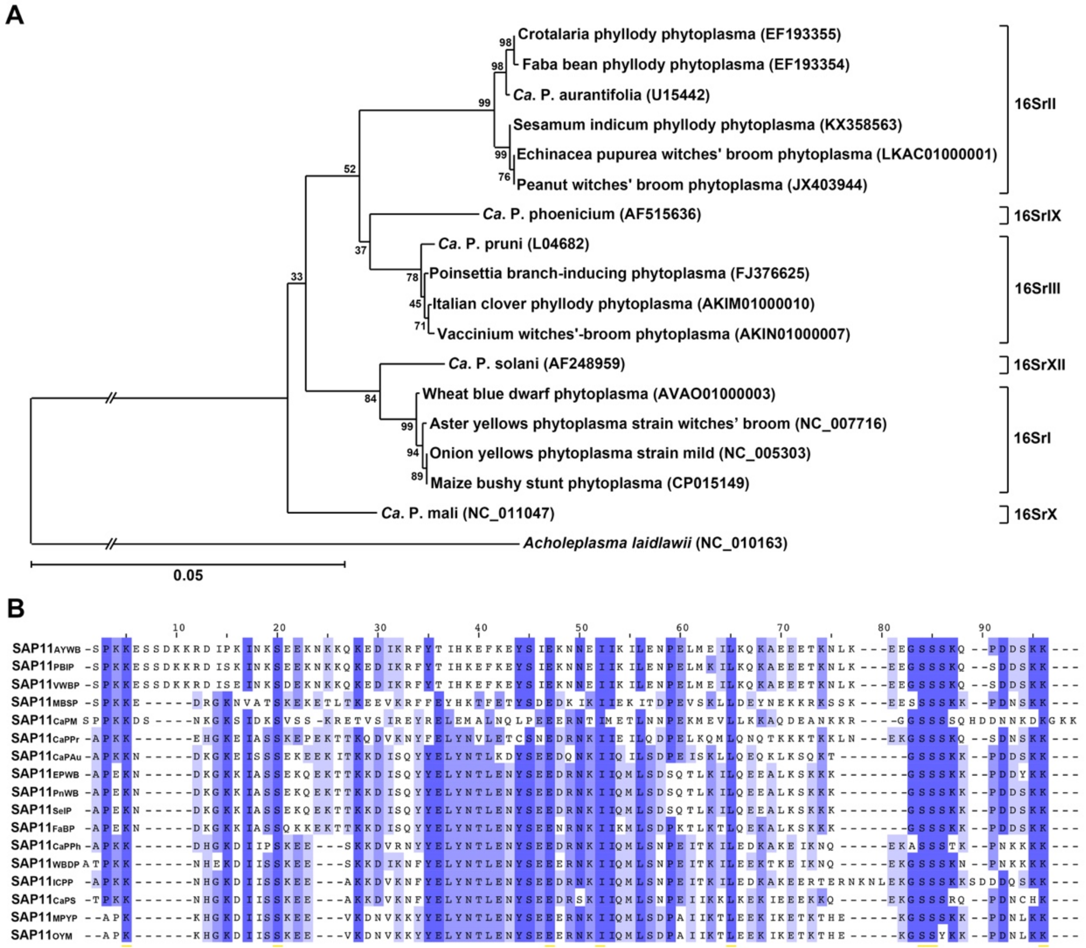

Fig. S1. Phylogenetic comparison and protein sequence alignment of phytoplasma 16Sr RNAs and SAP11 effectors. (**A**) A phylogenetic tree was constructed based on the comparison of 16S rRNA gene sequences of phytoplasmas containing putative SAP11 homologues. The neighbor-joining method followed by bootstrap analysis was used with MEGA 7.0 software. *A. laidlawii* served as an outgroup. The numbers at the branch points are bootstrap values that represent the percentages of replicate trees based on 1000 repeats. (**B**) Sequence alignments of SAP11 effectors without a signal peptide was constructed by MEGA 7.0 using clustalW and edited by Jalview software. Identical residues are shaded in blue, and the color gradient indicates the levels of sequence conservation at each position. Putative SAP11 homologues identified from Poinsettia branch-inducing phytoplasma (PBIP), Maize bushy stunt phytoplasma (MBSP), *Candidatus* Phytoplasma aurantifolia (CaPAu), Echinacea pupurea witches' broom phytoplasma (EPWB), Sesamum indicum phyllody phytoplasma (SeIP), Faba bean phyllody phytoplasma (FaBP), *Candidatus*

Phytoplasma phoenicium (CaPPh), and Malaysian periwinkle yellows phytoplasma (MPYP) were named $SAP11_{PBIP}$, $SAP11_{MBSP}$, $SAP11_{CaPAu}$, $SAP11_{EPWB}$, $SAP11_{SelP}$, $SAP11_{FaBP}$, $SAP11_{CaPPh}$, and $SAP11_{MPYP}$, respectively.

Supplementary Fig. S2

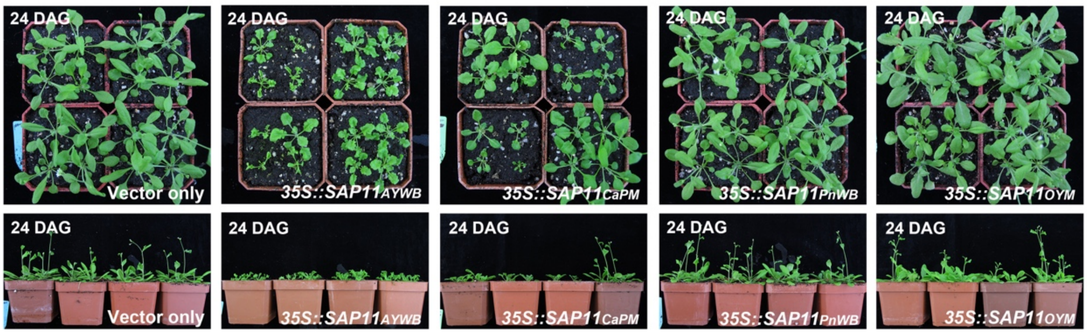

Fig. S2. Morphological comparison of independent *Arabidopsis* T1 transgenic plants. Morphological changes in independent *Arabidopsis* T1 transgenic plants expressing $SAP11_{AYWB}$, $SAP11_{CaPM}$, $SAP11_{PnWB}$, or $SAP11_{OYM}$ were compared against the morphological features of vector-only transgenic plants. Images were obtained at 24 days after germination (DAG).

Supplementary Fig. S3

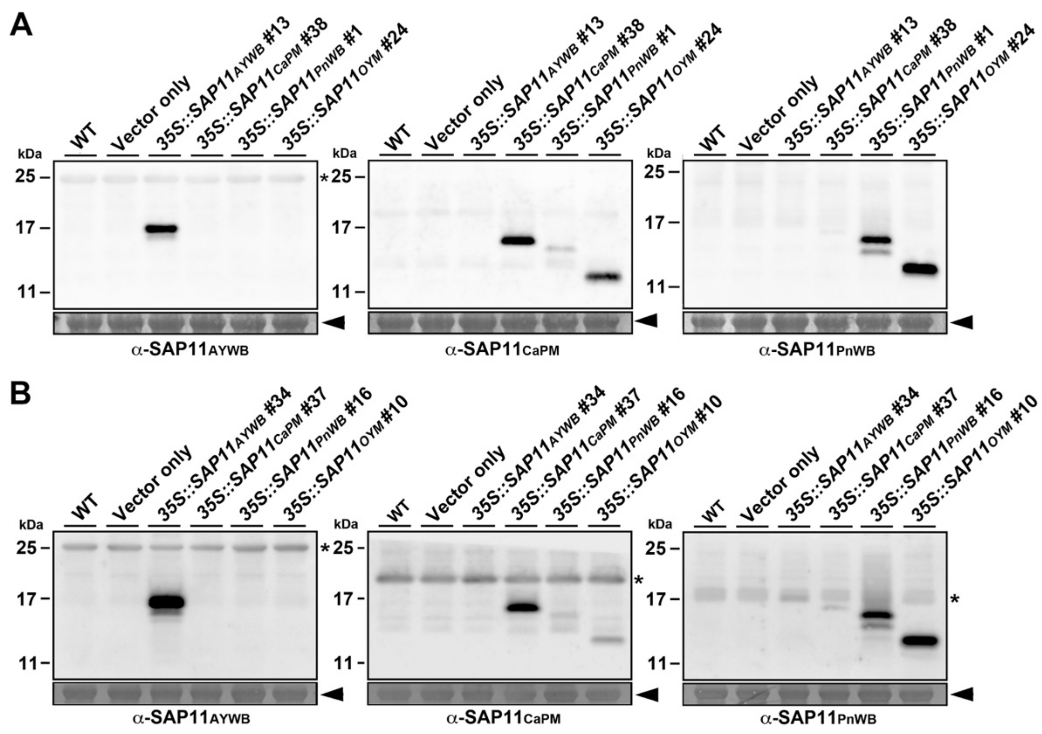

Fig. S3. Western blotting analysis of *Arabidopsis* homozygous transgenic lines expressing SAP11 effectors. The expression levels of SAP11 effectors were examined by western blotting using antibodies against $SAP11_{AYWB}$, $SAP11_{CaPM}$, and $SAP11_{PnWB}$. $SAP11_{OYM}$ could be recognized by α-$SAP11_{PnWB}$. Images were collected with a shorter exposure time (A) and a longer exposure time (B). As a loading control, the large subunit of RuBisCO visualized with Coomassie Brilliant Blue staining is indicated with the arrowhead. Non-specific bandings recognized by antibodies are indicated by asterisks.

Supplementary Fig. S4

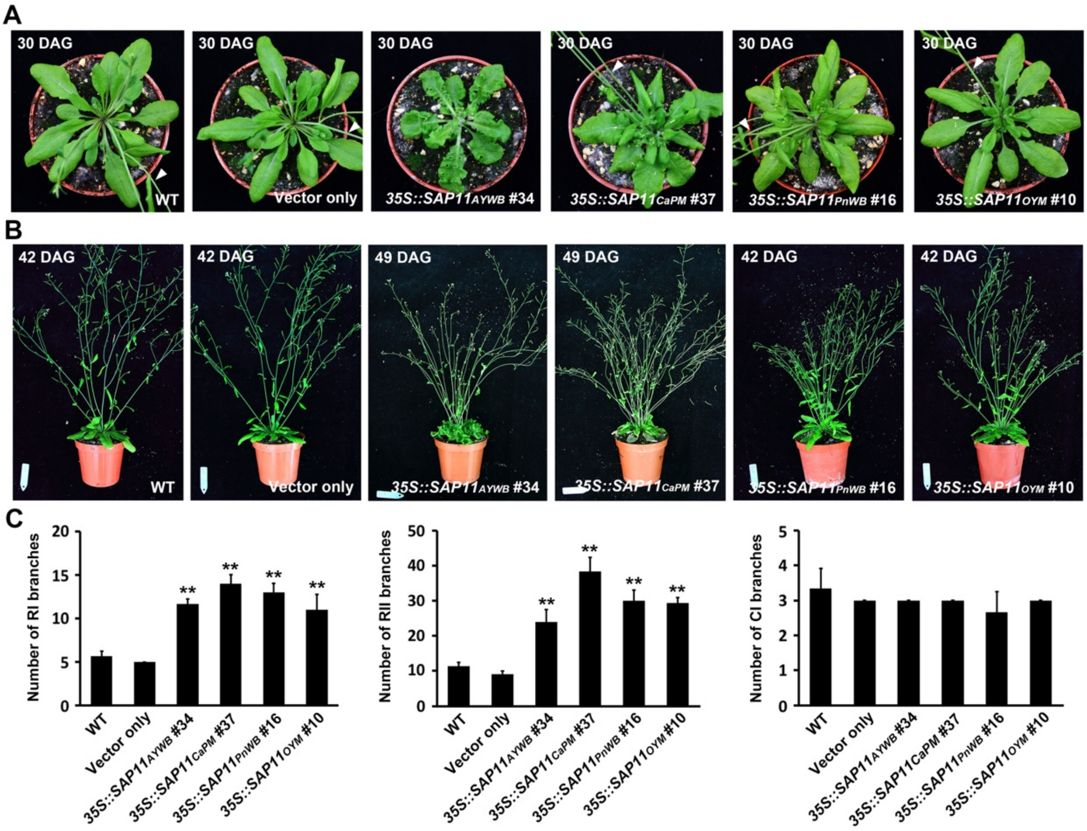

Fig. S4. Phenotypic comparison of rosette leaves and branching shoots of *Arabidopsis* homozygous transgenic lines. (**A**) The morphological changes in rosette leaves of *35S::SAP11* transgenic plants were compared against the morphological characteristics of wild-type (WT) and vector-only transgenic plants. Images were obtained at 30 DAG. Arrowheads indicate inflorescence. (**B**) Morphological changes in branching shoots of *35S::SAP11* transgenic plants were compared against the morphological characteristics of WT and vector-only transgenic plants. Images were obtained at 20 days after flowering. (**C**) The numbers of primary RI, RII, and CI branches of *35S::SAP11* transgenic plants were measured and compared with those of WT and vector-only transgenic plants. Statistically significant differences were determined using Student's *t* test (***P* < 0.01 for *35S::SAP11* transgenic plants versus vector-only controls).

Supplementary Fig. S5

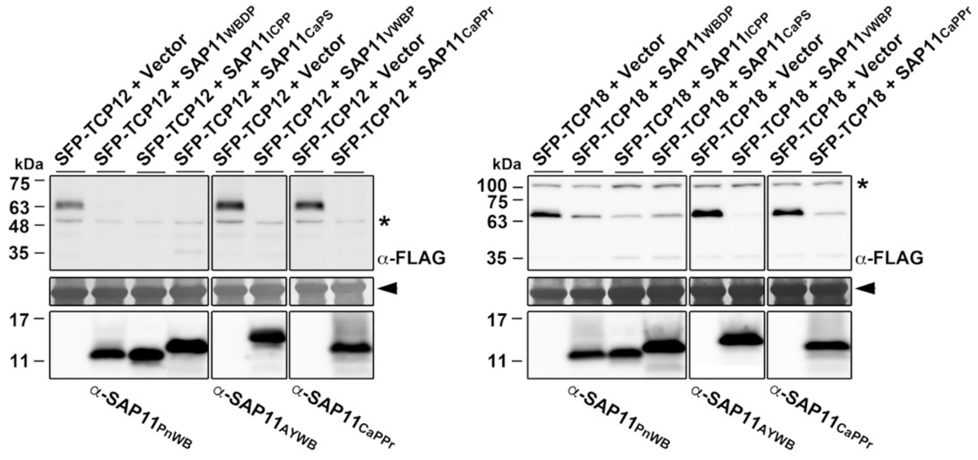

Fig. S5. Co-expression assays of SAP11 effector-mediated destabilization of class II TB/CYC-TCP transcription factors. Co-expression assays were conducted in *N. benthamiana* through agroinfiltration. The relative abundance levels of *Arabidopsis* TCP12 and TCP18 were examined in the presence of SAP11 effectors. Western blotting was conducted to examine the expression levels of FLAG-tagged TCPs (upper panel) and SAP11 effectors (lower panel) using a monoclonal antibody against FLAG tag and polyclonal antibodies against SAP11 effectors. $SAP11_{WBDP}$, $SAP11_{ICPP}$, and $SAP11_{CaPS}$ could be recognized by α-$SAP11_{PnWB}$, and $SAP11_{VWBP}$ could be recognized by α-$SAP11_{AYWB}$. As a loading control, the large subunit of RuBisCO visualized with Coomassie Brilliant Blue staining is indicated by the arrowhead (middle panel). Non-specific bandings recognized by antibodies are indicated by asterisks.

Supplementary Fig. S6

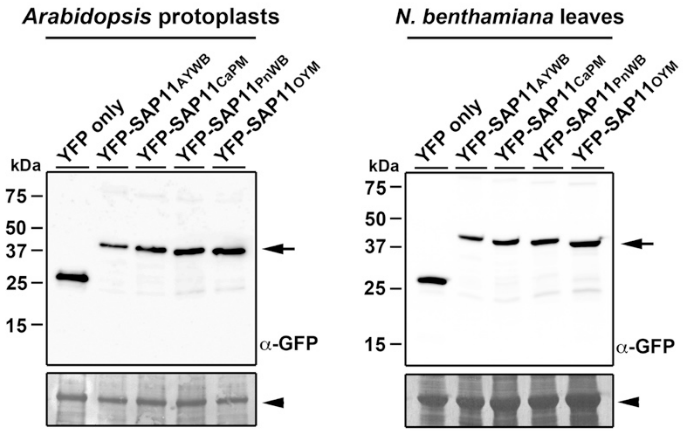

Fig. S6. Western blotting analysis of YFP and YFP-fused SAP11 effectors expressed in *Arabidopsis* mesophyll protoplasts and *N. benthamiana* leaves. N-terminal YFP-fused SAP11 effectors were transiently expressed under the control of a *35S* promoter. The relative abundance levels of YFP and YFP-fused SAP11 effectors in *Arabidopsis* mesophyll protoplasts and *N. benthamiana* leaves were examined by western blotting using an anti-GFP antibody (upper panel). As a loading control, the large subunit of RuBisCO visualized with Coomassie Brilliant Blue staining is indicated by the arrowhead (lower panel). The arrow indicates the YFP-fused SAP11 effectors.
